# *Pulsatilla vulgaris* Inhibits Cancer Proliferation in Signaling Pathways of 12 Reporter Genes

**DOI:** 10.3390/ijms24021139

**Published:** 2023-01-06

**Authors:** Grażyna Łaska, Elwira Sieniawska, Magdalena Maciejewska-Turska, Łukasz Świątek, David S. Pasco, Premalatha Balachandran

**Affiliations:** 1 Department of Agri-Food Engineering and Environmental Management, Bialystok University of Technology, 15-351 Bialystok, Poland; 2 Department of Natural Products Chemistry, Medical University of Lublin, 20-093 Lublin, Poland; 3 Department of Pharmacognosy with the Medicinal Plant Garden, Medical University of Lublin, 20-093 Lublin, Poland; 4 Department of Virology with SARS Laboratory, Medical University of Lublin, 20-093 Lublin, Poland; 5 National Center for Natural Products Research, Research Institute of Pharmaceutical Sciences, School of Pharmacy, University of Mississippi, University, MS 38677, USA

**Keywords:** saponins, phenolic acids, natural products, HeLa cells, luciferase reporter gene vectors

## Abstract

This study aimed to examine if methanolic extracts of *Pulsatilla vulgaris* Mill. can inhibit HeLa cell proliferation through the modulation of cancer-related signaling pathways. The cytotoxicity and chemical composition of *P. vulgaris* leaves and root extracts were also determined. Research showed that root extract of *P. vulgaris* inhibited 12 signaling pathways in a cervical cancer cell line and the most potent activation inhibition was observed for MYC, Notch, Wnt, E2F, Ets, Stat3, Smad, Hdghog, AP-1, and NF-κB, at a concentration of 40 µg/mL. The methanolic extracts of *P. vulgaris* enhanced apoptotic death and deregulated cellular proliferation, differentiation, and progression toward the neoplastic phenotype by altering key signaling molecules required for cell cycle progression. This is the first study to report the influence of *P. vulgaris* on cancer signaling pathways. Additionally, our detailed phytochemical analysis of the methanolic extracts of *P. vulgaris* gives a conclusion that compounds, which strongly suppressed the growth and proliferation of HeLa cancer cells were mainly triterpenoid saponins accompanied by phenolic acids.

## 1. Introduction

The *Pulsatilla* genus comprises 70 species, including 38 taxa in the Northern Hemisphere [1], with a long history of use in Traditional Chinese Medicine [2,3,4]. The biological and chemical diversity of the plants of the *Pulsatilla* genus identified them as a natural remedy for numerous ailments, including malaria, bacterial infections [4] or treatment of bronchitis, coughs, asthma, insomnia, hyperactivity, stress, anxiety, neuralgia, headaches, earache, and rheumatism [5]. Triterpenoid saponins, flavonoids, and phenolic acids were considered the predominant classes of specialized metabolites documented in aerial and underground parts of these plants [6].

In the present study, we focused on *Pulsatilla vulgaris* subsp. *vulgaris* Mill. (Pasque flower), a little-known representative of the family Ranunculaceae. Widespread in Central and Eastern Europe [7] *P. vulgaris* is classified as an extinct species (RE category) in Poland [8] due to agricultural intensification. Our previous paper reported the antifungal activity of this taxon. The extracts from *P. vulgaris* were active against the fungus *Candida glabrata* with the half-maximal inhibitory concentration (IC_50_) values of 11 μg/mL [9]. Previous phytochemical studies showed that *P. vulgaris* can be a potential source of bioactive compounds, such as triterpenoid saponins, mainly of the oleanane and lupane-type [10,11], which may have anticancer activity. The root methanolic extract from *P. vulgaris* showed the presence of Pulsatilla saponin D (hederagenin 3-O-α-l-rhamnopyranosyl (1→2)-[β-d-glucopyranosyl (1→4)]-α-l-arabinopyranoside) [12]. However other *Pulsatilla* species were much more explored for their antiproliferative activity. For example, the oleanane saponin from the methanolic extract of the root of *P. patens* subsp. *multifida* (G. A. Pritzel) Zämelis inhibited the growth of skin cancer [13]. Pulsatilla saponin A and Pulsatilla saponin B (oleanane and lupane-type, respectively), isolated from the root methanolic extract of *P. chinensis* (Bunge) Regel showed high cytotoxic activity against malignant lung cancer cells [14,15] and human liver tumor [16]. *P. chinensis* triterpene saponins have high antifungal [17], antiparasitic [18], antibacterial [19], antiprotozoal [20], and molluscicides [21] activities and are used in the treatment of inflammatory diseases, indigestion, premenstrual syndrome, and psychosomatic disorders [3]. Similarly, the saponin D from *P. koreana* Nakai inhibited the growth of cancer cells [22,23] and is used in the treatment of Alzheimer’s disease [24]. From the roots of *P. koreana,* 11 saponins were isolated, including five lupane-type, and one oleanane [25], which showed anticancer [26], antibacterial [27], antiparasitic [28], anti-inflammatory [29], antifungal and antibiotic activities [25]. The saponins produced by *P. chinensis* [14,15], *P. koreana* [23,25], *P. pratensis* (L.) Mill. [30], *P. nigricans* Storck [31], *P. cernua* (Thunb.) Bercht. et Opiz. [32,33], *P. turczaninovii* Kryl. et Serg. [34], *P. dahurica* (Fisch. ex DC.) Spreng. [35], show high biological activity not only as an anticancer [16,22,26] but also antioxidant [36], antimicrobial [37], immunomodulating [38], neuroactive [24,39,40], and cytotoxic [41,42].

In the present study, the cytotoxicity assessment of *P. vulgaris* extracts was performed using a panel of seven (normal and cancerous) mammalian cell lines after 48 h incubation in the neutral red assay [43,44] and the panel of two cell lines (VERO green monkey kidney, HeLa cervical adenocarcinoma) after 72 h incubation using the MTT-based assay [45,46]. Other *Pulsatilla* species and their active secondary metabolites have also been studied by many researchers. Flavonoids and anthocyanidins were found in the plant material of *P. slaviankae* (Zimmer.) Jordanov and Kožuharov, *P. montana* (Velen.) Zämelis and Paegle, *P. halleri* (Stoj. et Stef.) K. Krause, *P. cernua* [32,47,48], and *P. nigricans* [49], which were evaluated for the treatment of breast, colon, prostate cancer, and leukemia [50,51].

As can be seen from the above study of the literature, the species of *P. vulgaris* was not extensively studied. The first chemical composition reports of this taxon were in the 20th (1926–1927) and 40th (1949) years of the XX century [52,53,54], in which saponins, tannins [52,53], and anemonine drugs [54] were described. Research on this taxon is mainly focused on population ecology [7,55,56], life history [57], and life cycle biology [58,59] or molecular genetics [7,60]. *P.vulagris* is classified in the EU as a taxon “critically endangered” (CR category) in Austria [61], as “endangered” (EN category) in Germany [62] and Switzerland [63], and as species “near threatened“ by the International Union for Conservation of Nature [64]; therefore, it was rarely pharmacologically investigated. *P. vulgaris* is a “vulnerable” (VU category) species in the United Kingdom [65], Sweden [66], Slovakia, and Ukraine [67]; hence, the availability of this taxon in natural sites is low, also in Poland [8,68], what it is the cause that it is mainly cultivated artificially in Botanical Gardens and Greenhouses.

This is the first study to report a detailed phytochemical analysis of *P. vulgaris* extracts and the influence of this species on cancer signaling pathways, through which the growth and proliferation of HeLa cancer cells were strongly suppressed. For the evaluation of the activity of methanolic extracts from roots and leaves of *P. vulgaris*, we selected 13 luciferase reporter gene vectors. These vectors were represented by transcription factors, such as MYC, Ets, Notch, Wnt Stat3, Smad, AP-1, NF-κB, E2F, Hdghog, miR-21, k-Ras, FoxO, and pTK—control. Our study focused on the signal transduction in the presence of various inducers (IL-6, TGF-β, PMA, wnt-3a) when cells were exposed to extracts for 4–6 h. Additionally, a cytotoxicity assessment of *P. vulgaris* extracts was performed using a panel of seven (normal and cancerous) mammalian cell lines and a detailed phytochemical analysis of this species was completed, which allowed the identification of triterpenoid saponins and phenolic acids responsible for the antiproliferative activity.

## 2. Results and Discussion

The activity of *P. vulgaris* extracts in inhibiting HeLa cell proliferation in the signaling pathways of 13 reporter genes was described in this work. The cytotoxicity of the extracts was determined, and their chemical composition was described.

### 2.1. Cytotoxicity Studies

The roots and leaf extracts of *P. vulgaris* showed in vitro cytotoxicity to the mammalian cell lines in the neutral red assay (Table 1). The criteria for cytotoxicity evaluation of tested plant extracts were based on the guidelines set by the National Cancer Institute (NCI) and were as follows: CC_50_ < 20 μg/mL (high cytotoxic activity), CC_50_: 21–200 μg/mL (moderate cytotoxic activity), CC_50_: 201–500 μg/mL (weak cytotoxic activity), CC_50_ > 500 μg/mL (no cytotoxic activity) [69]. The P. vulgaris roots extract showed higher cytotoxicity, with the CC_50_ in the range of 39–42 μg/mL for normal mammalian cell lines and 31–57 μg/mL for cancer cell lines, than P. vulgaris leaves extract (CC_50_ 52–73 μg/mL). However, both extracts exerted a general cytotoxic activity (Table 1). Considering the above criteria, the methanolic extract of *P. vulgaris* can be categorized as moderately cytotoxic. The most potent inhibition of viability was observed for the HeLa cells; thus, this cancer cell line was selected for further analysis of P. vulgaris cytotoxicity and its influence on signaling pathways. However, it should be noted that Pulsatilla species producing Pulsatilla saponins (A, B, D) show general cytotoxic activity against the panel of different cancer cell lines [6,9,10,12,13,22,43,44].

The neutral red (NR) assay is a cell viability test based on the ability of live cells to take up the neutral red, which accumulates within the lysosomes of these cells. To further evaluate the cytotoxicity of *P. vulgaris* extracts, we have selected an alternative method, the microculture tetrazolium assay (MTT), which is based on the ability of cellular dehydrogenases to reduce the water-soluble tetrazolium salt to water-insoluble, purple formazan. The MTT assay is often used to determine cytotoxicity after exposure to toxic substances [45].

**Figure 1:**
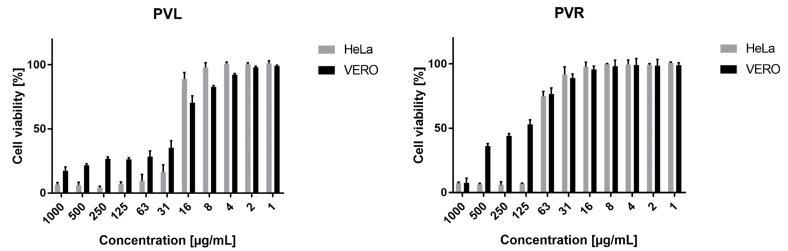
Cytotoxicity of *P. vulgaris* root (PVR) and leaf extracts (PVL) tested using MTT assay.

For the MTT test, we employed a longer incubation period of 72 h, and the results are presented in Figure 1, and the corresponding CC_50_ values are shown in Table 2. In the case of the PVR (*P. vulgaris* roots extract), the SI (selectivity index) value was 2.62, indicating selective anticancer activity. At the same time, the PVL (*P. vulgaris* leaves extract) showed similar cytotoxicity towards both normal and cancer cells. Interestingly, the PVR showed significantly lower toxicity towards VERO and HeLa when tested using MTT compared to the NR assay, whereas, for PVL, the MTT assay showed higher toxicity.

Studies showed that the NR and the MTT assay are among the most sensitive cytotoxicity assays showing statistically significant differences between the treated cells and the untreated controls. However, it was found that the results obtained using different methods are not always in agreement due to the diverse nature of each assay and the determination of different cellular functions [45,46,70]. Moreover, divergent results can be obtained when comparing these methods in assessing compounds’ toxicity on different cell lines [45].

For example, Fotakis and Timbrell [45] studied the cytotoxicity of cadmium chloride towards two hepatoma cell lines (HTC and HepG2) using various assays. After 24 h incubation, the cadmium chloride showed CC_50_ of 20 μM and 100 μM towards HTC cells using NR and MTT, respectively, which means that the NR indicated five times higher toxicity than the MTT. However, when HepG2 cells were used, the CC_50_ values obtained using NR and MTT were 8 μM and 15 μM, respectively, which indicated that NR showed less than two-fold higher toxicity than the MTT [45]. Triton X-100 showed similar toxicity towards mouse fibroblasts (L-M(TK-), ATCC CCL-1.3) using NR and MTT. Whereas, when chloroquine was tested, the NR indicated 100-fold higher toxicity than MTT because enzymatic assays like MTT may be influenced by enzyme inhibitors such as chloroquine [46]. Both NR and MTT assays use measurements of absorbance to calculate the number of viable cells after exposure to various xenobiotics, and both assays have their advantages but also significant disadvantages [70,71]. The NR assay indicates toxicity of substances that directly act on the cell membranes, while substances that damage metabolic pathways or interact with nuclear processes may not be adequately assayed. Thus, the NR is more suitable for predicting local irritation rather than systemic or delayed toxicity [71]. The MTT, by assessing the activity of cellular dehydrogenases, shows the disruption of a critical biochemical function and indicates early cytotoxicity in affected cells. However, it was shown that the mitochondria with active succinate dehydrogenase may remain in the dead cells, which leads to inappropriate interpretation as biologically active, surviving cells. Moreover, tested compounds may influence (enhance or reduce) succinate dehydrogenase activity without affecting the survival of the cells or may even directly interact with the MTT. The possibility of chemical reactions with MTT is particularly important when assessing the cytotoxicity of plant extracts since they are complex mixtures of compounds with versatile properties, including reductive activity, such as antioxidants [70]. Since there is no universal approach to cytotoxicity testing, using at least two assays based on the assessment of different parameters may be considered a good practice.

### 2.2. The Chemical Composition of the Studied P. vulgaris Methanolic Extracts

**Figure 2:**
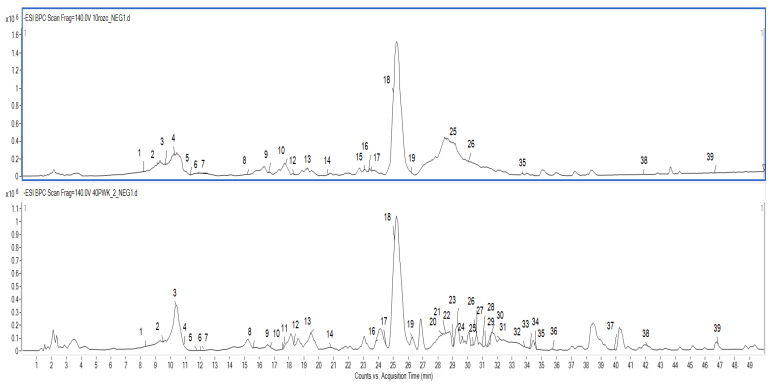
The base peak chromatograms of *P. vulgaris* aboveground parts (higher) and roots (lower) extracts. Numbers correspond to compounds described in Table 3.

In this study, methanolic extracts obtained from the roots and aboveground parts of *P. vulgaris* were subjected to sophisticated analysis involving a combination of chromatographic and spectroscopic methods (LC/ESI-QToF/MS-MS). As a result, **39** constituents were determined for the first time in *P. vulgaris* (Table 3 and Figure 2). Compounds presented in *P. vulgaris* samples were identified by comparing their UV spectra and high-resolution mass spectral data with relevant literature and publicly available databases such as HMDB (HMDB, https://hmdb.ca/, accessed on 4 May 2022) and PubChem (https://pubchem.ncbi.nlm.nih.gov, accessed on 4 May 2022). The identity of fourteen triterpenoid saponins, sixteen phenolic acids, four coumarins, one flavonoid, and three fatty acids was confirmed. The triterpenoid saponins and hydroxycinnamic acid derivatives represented the main class of specialized metabolites in *P. vulgaris*. However, similarly to previously reported data regarding *P. patens* constituents, a difference in phytochemical profile was observed depending on the analyzed plant part [6]. Saponins were distributed exclusively in the root samples. The MS data acquired in negative ionization mode are presented in Table 3 according to compounds elution order.

The saponin fraction is a distinct group of bioactive constituents distributed in various *Pulsatilla* species [12]. Regarding sapogenin skeleton constituents belonging to either oleanane- or lupine-type were identified, while the cleavage of the entire α-sugar chain or successive losses of single sugar fragments from C-28 or C-3, respectively, gave information about the position of substituents in oligoglycoside part [72,73]. Due to the similarity in the retention of chromatographic parameters and fragmentation mechanism of compounds **22**, **29**, and **33** to that described in our previous study, we identified them as 3-*O*-glucopyranosyl—arabinopyranosyl—23-hydroxybetulinic acid 28-*O*-rhamnopyranosyl(1-4)-glucopyranosyl(1-6)-glucopyranosyl ester, 3-*O*-arabinopyranosyl-23-hydroxybetulinic acid 28-*O*-rhamnopyranosyl(1→4)-glucopyranosyl(1→6)-glucopyranosyl ester and 28-*O*-rhamnopyranosyl (1-4)-glucopyranosyl (1-6)-glucopyranosyl 23-hydroxybetulinic acid ester, respectively. Compound **27** shared a similar fragment pathway to compound **22**. Compound **27** afforded the precursor ion at *m*/*z* 1205.5922 and the major fragment ion at *m*/*z* 735.3446. The mass difference between these two ions resulted from a typical neutral loss of 470 Da, supporting the substitution of two glucose and one rhamnose residue in the α-sugar chain. Moreover, the decrease of 30 Da of these two ions compared to that of compound **22** (*m*/*z* 1235.5926 and 765.4470) suggested the presence of one xylopyranosyl (−132 Da) rather than glucosyl group (−162 Da) linked to C-3 position. Therefore, according to [74], compound **27** was assigned to be 3-*O*-xylopyranosyl(1-2)-α-L-arabinopyranosyl-23-hydroxybetulinic acid 28-*O*-rhamnopyranosyl (1-4)-glucopyranosyl(1-6)-glucopyranosyl ester. Four of the analyzed compounds were classified as hederagenin-type saponins, namely 3-*O*-glucopyranosyl—arabinopyranosyl—hederagenin 28-*O*-rhamnopyranosyl(1-4)-glucopyranosyl(1-6)-glucopyranosyl ester (**24**), 3-*O*- arabinopyranosyl—hederagenin 28-*O*-rhamnopyranosyl(1-4)-glucopyranosyl(1-6)-glucopyranosyl ester (**31**), 3-*O*-arabinopyranosyl –28-*O*-glucopyranosyl hederagenin ester (**36**) and 3-*O*-arabinopyranosyl-28-*O*- arabinopyranosyl hederagenin ester (**37**). In the case of compound **37,** the generation of two intensive fragment ions at *m*/*z* 603.3976 and 471.3566 from adduct ion [M + HCOO]^−^ at *m*/*z* 781.4397 was presumed to be the result of sequential loss of one arabinose moiety (−132 Da) linked to C-3 and C-28 position. The fragment ions generated in MS/MS spectra for compound **28** might indicate Hederacoside C and Anemoside B4 structure; however, the insufficient data obtained does not allow for its unambiguous identification [72,74,75]. Similarly, in the case of compounds 30 and 34, the type of sapogenin skeleton was unknown, therefore compound **30** was proposed to be 3-*O*-rhamnopyranosyl-glucopyranosyl-hederagenin 28-*O*-rhamnopyranosyl(1-4)-glucopyranosyl(1-6)-glucopyranosyl ester or 3-*O*-rhamnopyranosyl-glucopyranosyl-23-hydroxybetulinic acid 28-*O*-rhamnopyranosyl(1-4)-glucopyranosyl(1-6)-glucopyranosyl ester, while compound **34** derivative of 23-hydroxybetulinic acid 28-*O*-rhamnopyranosyl(1-4)-glucopyranosyl(1-6)-glucopyranosyl ester or derivative of 28-*O*-rhamnopyranosyl(1-4)-glucopyranosyl(1-6)-glucopyranosyl hederagenin ester.

For compound **32**, the deprotonated molecular ion at *m*/*z* 1189.6050 was in accordance with the empirical molecular formula of C_58_H_94_O_25_. The fragments generated after CID (the collision-induced dissociation) were similar to that reported by [74], hence compound **32** was tentatively assigned as 3β-[(*O*-β-D-xylopyranosyl (1→2)-α-L-arabinopyranosyl) oxy]lup-20-(29)-en-28-oic acid 28-*O*-α-L-rhamnopyranosyl-(1→4)-*O*-β-D-glucopyranosyl-(1→6)-β-D-glucopyranosyl ester. Two compounds, **21** and **23**, annotated in roots only, were determined as 3-*O*-glucopyranosyl—arabinopyranosyl—bayogenin 28-*O*-rhamnopyranosyl(1-4)-glucopyranosyl(1-6)-glucopyranosyl ester and 3-*O*- arabinopyranosyl—bayogenin 28-*O*-rhamnopyranosyl(1-4)-glucopyranosyl(1-6)-glucopyranosyl ester, respectively, based on the presence of a specific fragment ion at *m*/*z* 487 in their MS/MS spectra, suggesting the bayogenin skeleton [6,12,73].

**Table 3:** The chemical composition of the studied aboveground (**L**) and root (**R**) *P. vulgaris* methanolic extracts.

Comp. No	Tentative Identification	Rt (min)	Molecular Formula	MW	[M − H]^−^	Fragments (*m*/*z*)	L	R	Ref
1.	Hydroxymelilotic acid	8.99	C_9_H_10_O_4_	182.0544	181.0544	163.0395; 149.0229; 135.0431; 119.0505	+	+	[6]
2.	Hydroxybenzoic acid isomer 1	9.560	C_7_H_6_O_3_	138.0317	137.0247	119.0112; 109.0319; 93.0344	+	+	
3.	Caffeic acid hexoside	10.382	C_15_H_18_O_9_	342.0951	341.0900	179.0305; 161.0221; 133.0258; 135.0419	+	+	
4.	Dihydroxycoumarin-*O*-glucoside	11.307	C_15_H_16_O_9_	340.0794	339.0750	177.0236 161.0324 133.0333	+	+	[76,77]
5.	Caftaric acid	12.582	C_13_H_12_O_9_	312.0481	311.0373	179.0345; 149.0084; 135.0476; 112.9942	+	+	[6,78]
6.	Dihydroxy-methoxycoumarin	12.260	C_10_H_8_O_5_	208.0340	207.0340	163.0403; 135.0434; 109.0296	+	+	[76,77]
7.	Tartaric acid	12.455	C_4_H_6_O_6_	150.0143	149.0143	149.0143; 121.0241; 87.0046; 72.9914	+	+	[76,78]
8.	Dihydroxycoumarin	15.653	C_9_H_6_O_4_	178.0266	177.0202	133.0280; 105.0343	+	+	[76,77]
9.	Caffeic acid	16.600	C_9_H_8_O_4_	180.0423	179.0322	135.0459	+	+	[6,78]
10.	Dihydroxy-methoxycoumarin-*O*-glucoside	17.706	C_16_H_18_O_10_	370.0900	369.0894	207.0318; 192.0059; 163.0046	+	+	[76,77]
11.	Coumaric acid hexoside	17.756	C_15_H_18_O_8_	326.0982	325.0982	265.0663; 235.0568; 205.0486; 163.0359; 145.0282	-	+	
12.	Vanillic acid	18.387	C_8_H_8_O_4_	168.0423	167.0338	152.0089; 108.0209	+	+	[6]
13.	Ferulic acid derivative	19.474	-	-	225.0790	193.0580; 161.0244; 135.0492	+	+	[76]
14.	Ferulic acid hexoside	20.733	C_16_H_20_O_10_	356.1107	355.1045	193.0522; 178.0223; 134.0387	+	+	
15.	Quercetin-*O*-deoxyhexoside-*O*-hexoside	23.391	C_27_H_30_O_16_	610.1534	609.1486	301.0351; 300.0194; 271.0257; 255.0332; 151.0051; 117.8824	+	-	[76]
16.	Hydroxybenzoic acid isomer 2	23.793	C_7_H_6_O_3_	138.0317	137.0261	93.0365	+	+	[6]
17.	Ferulic acid isomer	24.273	C_10_H_10_O_4_	194.0579	193.0522	161.0259; 134.0383	+	+	[6]
18.	Ferulic acid	25.276	C_10_H_10_O_4_	194.0579	193.0520	134.0424; 161.0307; 178.0260	+	+	[6,79]
19.	Ferulic acid di-hexoside	26.386	C_22_H_29_O_1_	518.1752	517.1752	355.1153	+	+	[80]
20.	Dicaffeoyltartaric acid isomer 1 (=Chicoric acid isomer 1)	28.361	C_22_H_18_O_12_	474.0798	473.0712	311.0373; 179.0310; 149.0059; 135.0430	+	+	[6,81]
21.	3-*O*-glucopyranosyl-arabinopyranosyl—bayogenin 28-*O*-rhamnopyranosyl(1-4)-glucopyranosyl(1-6)-glucopyranosyl ester	28.687	C_59_H_96_O_28_	1252.6044	1251.6044	781.4519; 619.3788; 487.3556; 471.0539; 469.1561	-	traces	[6,73]
22.	3-*O*-glucopyranosyl-arabinopyranosyl—23-hydroxybetulinic acid 28-*O*-rhamnopyranosyl(1-4)-glucopyranosyl(1-6)-glucopyranosyl ester	29.491	C_59_H_96_O_27_	1236.6139	1235.5926	765.4470; 603.3781; 469.1522	-	+	[6]
23.	3-*O*- arabinopyranosyl-bayogenin 28-*O*-rhamnopyranosyl(1-4)-glucopyranosyl(1-6)-glucopyranosyl ester	29.760	C_53_H_86_O_23_	1090.556	1089.5527	767.3829; 619.3829; 469.1502; 471.1573; 487.9452	-	+	[6]
24.	3-*O*-glucopyranosyl-arabinopyranosyl—hederagenin 28-*O*-rhamnopyranosyl(1-4)-glucopyranosyl(1-6)-glucopyranosyl ester	30.381	C_59_H_96_O_27_	1236.6139	1235.6016	765.4402; 603.3883; 469.1542; 367.1197; 471.4602; 451.1425	-	+	[6]
25.	Dicaffeoyltartaric acid isomer 2 (=Chicoric acid isomer 2)	30.586	C_22_H_18_O_12_	474.0798	473.0712	311.0399; 179.0327; 149.0062; 135.0412	+	+	[6,81]
26.	Caffeoyl feruloyltartaric acid	30.630	C_23_H_20_O_12_	488.0957	487.0957	325.0517; 293.0265; 193.0431; 179.0310; 149.0115; 135.0437	+	+	[81,82]
27.	3-*O*-xylopyranosyl (1-2)-α-L-arabinopyranosyl-23-hydroxybetulinic acid 28-*O*- rhamnopyranosyl (1-4)-glucopyranosyl (1-6)-glucopyranosyl ester	30.993	C_58_H_94_O_26_	1206.6033	1205.5922	735.3446; 603.4000; 469.1599	-	+	[74]
28.	Hederacoside C (3-O-arabinopyranosyl-rhamnopyranosyl-hederagenin 28-*O*-rhamnopyranosyl(1-4)-glucopyranosyl(1-6)-glucopyranosyl ester)/ Anemoside B4 (3-*O*-arabinopyranosyl-rhamnopyranosyl-23-hydroxybetulinic acid 28-*O*-rhamnopyranosyl(1-4)-glucopyranosyl(1-6)-glucopyranosyl ester)	30.993	C_59_H_96_O_26_	1220.619	1219.6033	749.4449; 603.4249; 469.1550; 471.0370	-	+	[74]
29.	3-*O*-arabinopyranosyl-23-hydroxybetulinic acid 28-O-rhamnopyranosyl(1→4)–glucopyranosyl (1→6)-glucopyranosyl ester	31.369	C_53_H_86_O_22_	1074.5611	1119.5586 [M + HCOO]^−^	1073.5510; 603.3963; 469.1590	-	+	[6]
30.	3-*O*-rhamnopyranosyl-glucopyranosyl-hederagenin 28-*O*-rhamnopyranosyl(1-4)-glucopyranosyl(1-6)-glucopyranosyl ester /3-*O*-rhamnopyranosyl-glucopyranosyl-23-hydroxybetulinic acid 28-*O*-rhamnopyranosyl(1-4)-glucopyranosyl(1-6)-glucopyranosyl ester	32.220	C_60_H_96_O_27_	1248.6204	1247.6204	1187.6022; 777.4205; 469.1467	-	+	
31.	3-*O*-arabinopyranosyl—hederagenin 28-*O*-rhamnopyranosyl(1-4)-glucopyranosyl(1-6)-glucopyranosyl ester	33.829	C_53_H_86_O_22_	1074.5611	1073.5628	603.4048; 471.1944; 469.1651; 409.1363	-	+	[6]
32.	3β-[(*O*-β-D-xylopyranosyl (1→2)-α-L-arabinopyranosyl) oxy]lup-20-(29)-en-28-oic acid 28-*O*-α-L-rhamnopyranosyl-(1→4)-*O*-β-D-glucopyranosyl-(1→6)-β-D-glucopyranosyl ester	34.267	C_58_H_94_O_25_	1190.6084	1189.6050	719.4455; 587.3973; 469.1608	-	+	[74]
33.	28-*O*-rhamnopyranosyl(1-4)-glucopyranosyl(1-6)-glucopyranosyl 23- hydroxybetulinic acid ester	34.502	C_48_H_78_O_18_	942.5223	987.519 [M + HCOO]^−^	941.4754; 471.3436; 469.1527	-	+	[6]
34.	derivative of 23-hydroxybetulinic acid 28-*O*-rhamnopyranosyl(1-4)-glucopyranosyl(1-6)-glucopyranosyl ester/ derivative of 28-*O*-rhamnopyranosyl(1-4)-glucopyranosyl(1-6)-glucopyranosyl hederagenin ester	34.502	-	1027.5420	1026.5420	941.5067; 471.3329; 469.1383	-	+	
35.	Trihydroxy-octadecenoic acid	35.697	C_18_H_34_O_5_	330.2292	329.2292	293.2082; 229.1428; 211.1264; 171.0929	+	+	[76]
36.	3-*O*-arabinopyranosyl–28-*O*-glucopyranosyl hederagenin ester	39.886	C_41_H_66_O_13_	766.4503	811.4472 [M + HCOO]^−^	765.4380; 603.3835; 471.3425	-	+	[6]
37.	3-*O*-Arabinopyranosyl –28-*O*-arabinopyranosyl hederagenin ester	42.089	C_40_H_64_O_12_	736.4486	781.4397 [M + HCOO]^−^	735.4486; 603.3976; 471.3566	-	+	
38.	Fatty acid	46.663	C_18_H_30_O_3_	294.2175	293.2175	275.1987; 224.1362; 195.1427; 171.0968	tr	+	[76]
39.	Fatty acid	48.944	C_18_H_32_O_3_	296.2344	295.2334	277.2158; 195.1374; 177.1248	tr	+	[76]

In terms of phenolic acids, we characterized **sixteen** of them in both roots and leaves samples. Only four were classified as hydroxybenzoic acids (**1**,**2**,**12**,**16**), while the remaining majority belonged to the hydroxycinnamic acid derivatives. Among them, the most intense peak recorded in the MS spectrum belonged to compound **18** with deprotonated molecular ion at *m*/*z* 193.0520 and three abundant product ions at *m*/*z* 178.0260, 161.0307, and 134.0424. By comparison with the reference standard, compound **18** was confirmed to be ferulic acid, while compound **17**, eluted earlier, was assigned as a ferulic acid isomer [83]. In addition, several ferulic acid derivatives were noticed between 19–27 min (compounds **13**, **14**, **19**). Caffeic acid (**9**) and its hexoside (**3**) were identified based on specific caffeic acid fragment ions observed in MS/MS spectrum, resulting from molecule decarboxylation and dehydration [79,84]. Four cinnamyl tartaric acid esters were also noticed, such as caftaric acid (**5**), caffeoyl-feruloyltartaric acid (**26**), chicoric acid isomer 1 (**20**), and isomer 2 (**25**) [85]. Caffeoyl-feruloyltartaric acid previously reported in various parts of *Cichorium intybus* L. was identified in *P. vulgaris* for the first time [81,82]. Compound **11,** tentatively identified as coumaric acid-hexoside, based on typical for coumaric acid fragment ions at *m*/*z* 163.0359 and 145.0282, was observed exclusively in roots, whereas quercetin-*O*-deoxyhexoside-*O*-hexoside (**15**), the only one flavonoid found, was annotated only in leaves. Four compounds **4**, **6**, **8**, and **10** detected between 10–18 min in both roots and leaves were classified as coumarins. However, their profile differs from coumarins found in other *Pulsatilla* species [86,87]. Based on the calculated molecular formula and the characteristic for coumarins, loss of small units (CO, CO_2_, H_2_O) analyzed compounds were tentatively assigned to be dihydroxycoumarin-*O*-glucoside (**4**), dihydroxy-methoxycoumarin (**6**), dihydroxycoumarin (**8**) and dihydroxy-methoxycoumarin-*O*-glucoside (**10**) [77].

### 2.3. The Cancer Activity of P. vulgaris in Signaling Pathways in HeLa Cells

**Figure 3:**
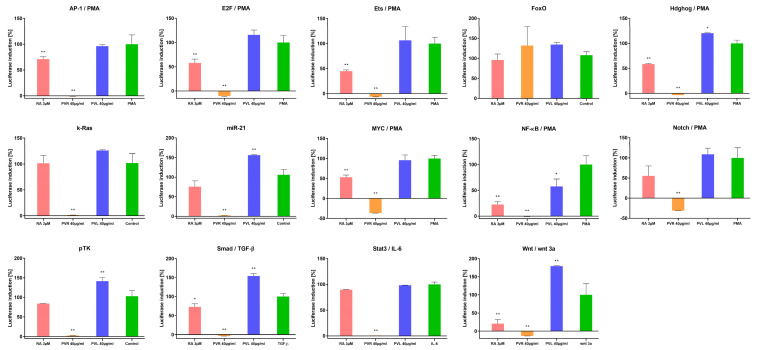
The inhibition of activations mainly of Notch, MYC, E2F, Wnt and NF-κB, Ap-1, Smad, Hdghog, Ets, Stat3 by methanolic extract of *Pulsatilla vulgaris* and reference/control compound—resveratrol analog. IL-6, TGF-β, PMA, wnt 3a—Inducer (promoter) of cancer processes. Resveratrol analog (RA)—the analog 3,5,4′-trihydroxy-*trans*-stilbene, *trans*-resveratrol or (*E*)-resveratrol—is a stilbenoid, a type of natural phenol, and a phytoalexin with anticancer activity. PVR—*Pulsatilla vulgaris* root extract (40 µg/mL); PVL—*Pulsatilla vulgaris* leaves extract (40 µg/mL); PMA—Phorbol 12—myristate 13—acetate; TGF-ß—Transforming growth factor beta; IL-6—Interleukin 6; wnt 3a—Wnt family member 3A. The obtained data were statistically analyzed using GraphPad Prism (two-way ANOVA, Dunnett’s multiple comparisons test), where * *p* < 0.05; ** *p* < 0.01.

**Figure 4:**
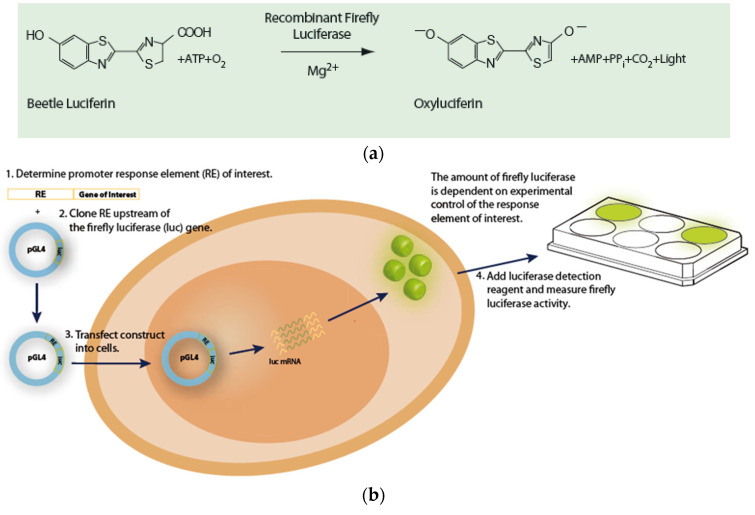
The reaction catalyzed by Firefly (*Photinus pyralis)* luciferase (**a**) and treatment of transfected cells and measurement of biological stimulation by determining the amount of active luciferase (**b**). Reprinted with permission from Ref. [90]. Copyright 2008, Promega Corporation.

The antiproliferative activity of *P. vulgaris* extracts was assessed using a panel of 13 luciferase reporter gene vectors (Figure 3, Tables S1 and S2). The luciferase expression within each vector was driven by enhancer elements (inducers, promoters, such as IL6, TGF-β, PMA, and Wnt 3a) that bind to specific transcription factors [88]. In this study, the genus of firefly luciferase (*Photinus pyralis*, *Pp Luc*) was used. *Pp Luc* catalyzes the conversion of ATP, luciferin, and O_2_ into the products: AMP, PP_i_, CO_2_, H_2_O, oxyluciferin and light with a wavelength of 562 nm (Figure 4a). Firefly luciferase is in the form of a monomer, and its efficiency is much higher than that of bacterial luciferase (the marine bacteria *Vibrio fischeri* and *Vibrio harveyi* (lux) or the *Renilla reniformis* (sea pansy (Rr Luc) [89].

The results showed that the methanolic extract from the roots of *P. vulgaris* was more potent than the leaf extract of this species in inhibiting the activation of twelve pathways analyzed, except for only one, FoxO (Forkhead proteins) (Figure 3).

In Figure 3, the numerical value expresses the percentage ratio of the activity of the test samples to the activity of the tumor promoter determined for the protein encoded by the luciferase reporter gene. The lower the numerical value of the data, the activity of the test sample was greater in inhibiting Hela cell proliferation than that of the tumor promoter. The active compounds of methanolic extract from the roots of *P. vulgaris* were found to strongly inhibit the activation of Stat3, Smad, AP-1, NF-κB, E2F, MYC, Ets, Notch, Wnt, Hdghog, pTK—control, miR-21 and k-Ras signaling (Figure 3). Each of the apoptotic signaling mediators was inhibited stronger by compounds from root extract than from leaf extract. The methanolic extract from the roots of *P. vulgaris* was stronger in potency than the active antitumor compound resveratrol analog in each concentration studied for inhibition of signaling. The only exception was the apoptotic mediator FoxO, where the resveratrol analog was more effective than active compounds from both of extracts of *P. vulgaris*. The transcriptional activity of FoxO controls cell proliferation and apoptosis and regulates the cell cycle and apoptotic genes such as the cyclin-dependent kinase inhibitor (CKI) p27(KIP1) [91,92], Fas ligand [93], Bcl-6 [94] and Bim [95,96].

The active compounds of methanolic extract from the leaves of *P. vulgaris,* similarly to the resveratrol analog, inhibited the activation of Stat3 and k-Ras, but significantly less than it inhibited the activation of Wnt, NF-κB, MYC, Notch, Ets, Smad, Hdghog, Ets, pTK—control and AP-1 signaling (Figure 3).

The statistical analysis showed that the largest statistically significant differences for the methanolic extract from the roots of *P. vulgaris* were in inhibiting the activation of Notch, Myc, E2F, and Wnt. These transcription factors (except Wnt) were activated by phorbol 12-myristate-13-acetate, a protein kinase activator (PMA) (Figure 3). The Notch can also be activated by a specific serine/threonine (Ser/Thr) protein kinase [97], MYC by—the direct interaction with the acetyltransferases p300, and CBP [98], and E2F by—cyclins, cyclin-dependent kinases CDKs connected with the cyclin-dependent kinase inhibitors (pp. 15–16) [99].

Notch promotes proliferation signaling during neurogenesis, and its activity is inhibited by neuronal differentiation and tumor cell proliferation [97]. Notch signaling plays a vital role in regulating embryonic development and cellular processes during development and adult tissue renewal. It was found that the modulation of the Notch pathway is essential in controlling the fate of “cancer stem cells”. Mammalian cells contain four Notch receptors, the activity of which is used to study pathway dynamics in a normal and disease context. Factors that are inhibitors of Notch are being investigated as potential agents for the treatment of cancer [100].

In studies of the expression of cancer-related genes, it was found that abnormal expression of oncogenes may be responsible for carcinogenesis. Therefore, MYC (induced nuclear protein antigen) and other members of the proto-oncogene family (c-, L-, and N-myc) are central regulators of cell growth, and their deregulated expression is associated with many cancers [98]. Myc encoded by the MINA gene show elevated expression in up to 70% of all human malignancies. MYC is a pleiotropic transcription factor that controls the mediators of apoptosis and directly regulates target genes responsible for the proliferation and growth of cancer cells [101].

E2F are proteins from the family of transcription factors that are involved in regulating the cell cycle [99]. This family includes activators (E2F1, E2F2 and E2F3a) and inhibitors (E2F3b, E2F4-8). Among E2F transcriptional targets are cyclins, CDKs, replication proteins, and DNA repair. Some human tumors have concurrent tumor cell inactivation and E2F amplification and overexpression. That is why researchers propose that there are alternative tumor-promoting activities for the E2F family, which are independent of cell cycle regulation [99].

The WNT family of genes consists of structurally related genes encoding the secretion of proteins in signaling pathways involved in tumor formation. WNT gene clusters are recombination hot spots associated with carcinogenesis [102]. Among 19 WNT genes, the WNT3A gene is clustered in human chromosome 1q42, where during carcinogenesis, recombination results in chromosomal translocation, gene amplification, and deletion. Protein Wnt-3a is encoded by the WNT3A gene and might play key roles in the maintenance of cells in the undifferentiated proliferation stage through activation of the β-catenin—TCF signaling pathway [103].

In this research, it was found that methanolic extract from the root of *P. vulgaris* had a similar effect on the inhibition of HeLa cell proliferation in signal transduction pathways driven by the minimal thymidine kinase promoter (pTK—control) and by the transcription proteins miR-21 and K-Ras. The other signaling pathways were more significantly inhibited. MicroRNA 21 (miR-21), is a signaling pathway in the cell involved in the regulation of proteoglycans in cancer [104]. It is an RNA gene related to a class of microRNA (miRNA) genes active in the post-transcriptional regulation of gene expression, including mRNA stability and translation [105]. The k-Ras is a protein mainly involved in the regulation of cell division [106].

**Figure 5:**
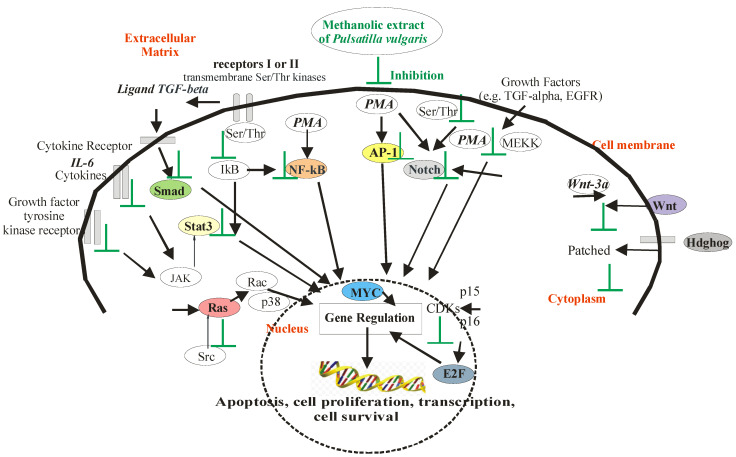
Oncogenic signaling pathways targets of *Pulsatilla vulgaris* extract. Mechanism of extract action in HeLa cells. The methanolic extracts of *Pulsatilla vulgaris* enhance apoptotic death, deregulated cellular proliferation, differentiation, and progression towards the neoplastic phenotype by altering key signaling molecules required for cell cycle progression [117,118,119]. Colors in the figure: Green captions–plant extract and inhibition mechanism; red captions–cell and its elements; Colors of transcription proteins: green–Smad, sand–Stat3 (Signal transducers and activators of transcription), yellow–AP1 (Activator protein 1), pink–Ras (Ras family kinases), violet–Wnt (WNT gene family), light grey–Notch (Neurogenic locus notch homolog protein), gray–Hdghog, blue–MYC (induced nuclear protein antigen), graphite–E2F protein, light brown–NF-κB (nuclear factor kappa-light-chain-enhancer of activated B cells); White colors of protein kinase: PMA (protein kinase activator), Ser/Thr (serine/threonine protein kinase), JAK (Janus kinase), MEKK (MAP kinase), IkB (Inhibitor of kB), Src (Protooncogene tyrosine-protein kinase), Rac (Subfamily of the Rho family of GTPases), p38 (mitogen-ctivated protein kinases), Wnt-3a protein; Gray color through cell membrane–receptors–Receptors I or II (transmembrane Ser/Thr kinases), Cytokine receptors–cytokines IL-6, Ligand TGF-beta, Growth factor tyrosine kinase receptor, EGFR–Epidermal growth factor receptor, Patched (protein patched homolog).

Stronger modulators of signaling pathways than pTK (control) against NF-κB, AP-1, Smad, Hdghog, Ets, and Stat3 were active compounds of methanolic extract from the roots of *P. vulgaris* (Figure 3). In our study, NF-κB (nuclear factor kappa-light-chain-enhancer of activated B cells) was activated by PMA (protein kinase activator). NF-κB can also be triggered by the enzyme IκB kinase and then translocated into the nucleus. NF-κB, a protein complex from the family of transcription factors, is present in the cytoplasm of each cell, where it plays a vital role in regulating the immune response to infection, and disturbances in its regulation are associated with tumors [107,108]. Activator protein 1 (AP-1), as a transcription factor in gene expression, controls proliferation, differentiation, and apoptosis and regulates factors of stress and growth, cytokines, and viral and bacterial infections [109]. In our study, Smad activated by TGF-β in the signaling pathway was connected with type I and type II receptors, which are transmembrane Ser/Thr kinases. Smad complexes accumulate in the nucleus and regulate target gene expression [110]. Therefore, they are essential in signaling and regulating cell functions during the whole life of the organism. Hdghog (synonyms: Hedgehog-Patched (Hh-Ptch), Hedgehog-Patched-Smoothened (Hh-Ptch-Smo)), in our modulation of cancer-related signaling pathways was activated by PMA (Figure 3). It is a transcription factor regulated by protein kinases, responsible for signal conveyance from the cell membrane into the nucleus (Figure 5). The Hdghog signal transduction pathway is important and was recently noted in the development of cancers in various organs, such as the brain, lung, mammary gland, prostate, and skin. Basal cell carcinoma, the most common form of cancerous malignancy, has the closest association with hedgehog signaling [111]. The Ets in our signaling pathways were activated by PMA, too (Figure 3). ETS (erythroblast transformation specific) belongs to the largest families of transcription factors to be associated with cancer [112]. Ets is a transcriptional activator that through changes in gene expression and their deregulation leads to carcinogenesis (cancer, tumor formation) and apoptosis (programmed cell death) [113]. It is also a key factor in angiogenesis, i.e., the neoplastic ability to form blood vessels within a neoplastic tumor that causes the tumor to further increase in mass [114]. In our study, STAT3 (signal transducers and activators of transcription) was activated by cytokine IL-6, which leads to its activation of Janus kinase (JAK) (Figure 5). Stat3 is phosphorylated by the receptor [115] and translocated to the nucleus, where it promotes prooncogenetic genes [116]. The inhibition of STAT3 in HeLa cell signaling caused by the methanolic extract of *P. vulgaris* is associated with the inhibition of growth and cell proliferation, and with apoptosis (Figure 5).

**Figure 6:**
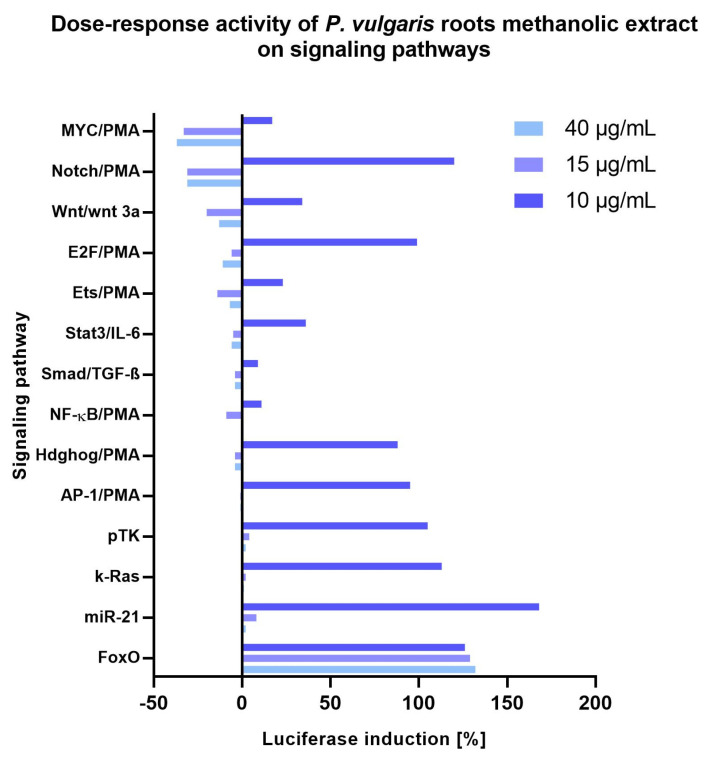
The active compounds from the roots methanolic extract of *P. vulgaris* against cancer−related signaling pathways in HeLa cells with different concentrations of 40 µg/mL, 15 µg/mL, and 10 µg/mL.

It was found that active compounds of methanolic extract from the root of *P. vulgaris* were more potent than those from the methanolic extract of leaves. We have also determined the dose–response influence of this extract on the cancer-related signaling pathways. *P. vulgaris* root extract at a concentration of 40 µg/mL was more potent in inhibiting the activation of six signaling pathways (Stat3, E2F, MYC, pTK, miR-21, and k-Ras), except only one—FoxO (Figure 6). At the concentration of 15 µg/mL, stronger activation of three cancer-related signaling pathways (NF-κB, Ets, Wnt) and the same influence on four signaling pathways (Smad, AP-1, Hdghog, and Notch) was observed. The *P. vulgaris* root methanolic extract at 10 µg/mL showed the lowest influence on all signaling pathways (Figure 6).

This is the first report describing the activity of constituents of methanolic extracts of *P. vulgaris* against different 13 signaling pathways of the cervical cancer-related HeLa cells. The vectors and inducers used in this study to assess the activity of the cancer-related signaling pathways were previously reported by other authors [44,86]. Similarly, resveratrol [(E)-3,5,4′-trihydroxystilbene], the parent compound of the analog used in these studies as a positive control, is used as a potential modulator of signal transduction pathways for cancer and carcinogenic response also in studies by other authors [44].

The performed analysis allowed us to tentatively identify fourteen triterpenoid saponins, sixteen phenolic acids, four coumarins, one flavonoid, and three fatty acids, which can contribute to the observed activity of *P. vulgaris*. Additionally, other plant secondary metabolites were studied for the inhibition of cancer-related signaling pathways [6,120]; however, their mechanism of action was related to induction of the oxidative stress in cells. In that way, they target redox reactions, and redox-sensitive cysteine [121] and additionally play a major role in reactive oxygen species (ROS) generation and the depletion of the antioxidant glutathione (GSH) [122].

The transcription factors (Stat3, Smad, AP-1, NF-κB, E2F, MYC, Ets, Notch, FoxO, Wnt, Hdghog, miR-21, k-Ras) play important roles in cancer-related signaling pathways by regulating cytological processes such as differentiation, cell death, cellular proliferation, behavior, oncogenic transformation and apoptosis [123]. Among cancer-associated genes and driver gene mutations identified, the vast belong to approximately 13+ different signal transduction pathways [6,44,86]. In our research, these vectors were used as signaling nodes for oncogenic pathways, which transduce intracellular and extracellular signals to the nucleus and control the expression of genes [124,125] responsible for physiological processes such as cell growth, proliferation, differentiation, positioning, migration, metabolism, and apoptosis [126,127]. The research found that uncontrolled cell division results from gene mutations in DNA, leading to unlimited proliferation (multiplication) of cancer cells and neoplastic processes (neoplasia, carcinogenesis) [128].

Herein, we report that *P. vulgaris* inhibits all of the tested signaling pathways with significantly higher potency than previously studied *P. patens* [6]. For example, in the case of k-Ras and Notch, the *P. patens* showed induction of luciferase of approx. 60%, whereas *P. vulgaris* completely inhibited those pathways. The action of methanolic extract *P. vulgaris* is based on the inhibition of mitosis in the HeLa cell cycle (Figure 5). The active compounds of *P. vulgaris* start the induction of apoptosis and cause deregulated cellular proliferation, which leads to apoptotic death of the HeLa cells and inhibition of the growth of cancer cells. The suggested influence of *P. vulgaris* on apoptosis was based on the studies of cancer-related signaling pathways in HeLa cells, being the primary goal of our research, and not on the direct measurements of apoptosis. We acknowledge that the proapoptotic activity of *P. vulgaris* requires additional research, such as measurements of cellular caspases (caspase-3, -7, -8 or -9) related to apoptosis, studies of gene mutations in DNA and DNA fragmentation and biological tests using fluorescence microscopy or flow cytometry. Our future scientific endeavors will focus on the isolation of bioactive molecules responsible for the observed influence on signaling pathways and detailed studies of their pro-apoptotic activity.

## 3. Materials and Methods

### 3.1. Plant Material

The leaves and roots of four years flowering individuals *P. vulgaris* subsp. *vulgaris* Mill. were collected in May 2016–2018 from cultivation conducted in the Podlasie Botanical Garden “The Herbal Corner”, Podlaskie Province, in Northeastern Poland. Plant material was identified by Prof. Grażyna Łaska from the Bialystok University of Technology. In Poland, the species *P. vulgaris* is currently extinct in Poland on natural sites [68]. It was only reported at four sites on the habitats of the pine forests before 1930 [129,130]. In June 2018 the plant material in the form of dry roots (59.3 g) and leaves (28.1 g) was extracted by accelerated solvent extraction (ASE) technique (SpeedExtractor E-916, Buchi) with 80% methanol. The extraction time was 30 min, the temperature was 100 °C and the pressure was 120 bar. After the evaporation of methanol using a rotor evaporator, a plant extract was obtained from roots (1.1 g) and leaves (0.95 g)

### 3.2. LC/ESI-QTOF-MS Procedure

The *P. vulgaris* samples were analyzed qualitatively by LC-ESI-QTOF-MS system composed of an Agilent 1200 Infinity chromatograph hyphenated to a 6530B Accurate-Mass Q-ToF mass spectrometer (Agilent Technologies, Santa Clara, CA, USA). After the chromatographic separation of *P. vulgaris* components on a Gemini^®^ column (3 μm i.d. C18 with TMS endcapping, 110 Å, 100 × 2 mm), the detailed qualitative profiling was performed using soft, electrospray ionization (ESI) in negative mode. The LC/MS method parameters followed our previous report [131]. The MS data acquisition was performed using the MassHunter software (Agilent Technologies). The identification was carried out based on the UV and mass spectra obtained, in comparison with the fragmentation behavior of these compounds reported in scientific literature and records available in HMDB [78] and PubChem [76] databases.

### 3.3. Cell Cultures and Media

Cytotoxic activity in the laboratory of the National Center for Natural Products Research (NCNPR) of the University of Mississippi (MS, USA) was determined against five human cancer cell lines (HeLa, SK-OV-3, KB, SK-MEL, BT-549) and two noncancerous kidney cell lines (LLC-PK1 and VERO) (see Table 1). All cell lines were obtained from the American Type Culture Collection (ATCC, Rockville, MD, USA).

Separately, the cytotoxicity of *P. vulgaris* extracts was assessed against HeLa and VERO cell lines after 72 h incubation using the MTT-based assay. The VERO (ECACC, No. 84113001) and HeLa (ECACC, No. 93021013) cells were grown in T25 flasks and 96-well plates using MEM (Corning, Tewksbury, MA, USA). Cell media were supplemented with fetal bovine serum (FBS, Biowest) and antibiotics (Penicillin-Streptomycin Solution, Corning). Cells were passaged using media supplemented with 10% FBS, whereas the media used for experiments contained 2% serum only. All experiments with cell cultures were conducted at 37 °C in the 5% CO_2_ atmosphere (CO_2_ incubator, Panasonic Healthcare Co., Ltd., Tokyo, Japan). The DMSO (dimethyl sulfoxide) and 3-(4,5-dimethylthiazol-2-yl)-2,5-diphenyltetrazolium bromide (MTT) were purchased from Sigma, whereas phosphate-buffered saline (PBS) and trypsin were from Corning.

### 3.4. Cytotoxicity Testing

The panel of healthy and cancer cell lines (in vitro) for cytotoxicity of root and leaf extracts *P. vulgaris* and cell viability were determined by the neutral red method [43,44] in the NCNPR, University of Mississippi (University, MS, USA). The cytotoxicity of extracts was evaluated using the CC_50_ values and was tested for the viability of healthy cell lines (kidney fibroblast, kidney epithelial cells) and cancer cell lines (cervical carcinoma, ovarian carcinoma, epidermal carcinoma, skin melanoma, breast carcinoma) (see Table 1). In this assay, we used 96-well tissue culture-treated plates. The cells were seeded at a density of 25,000 cells/well and grown for 24 h. In the next step, samples of extract of *P. vulgaris* at different concentrations were added and cells were further incubated for 48 h. CC_50_ values were obtained from dose–response curves of percent growth inhibition against test concentrations. Doxorubicin was used as the control drug (a positive control), while DMSO was used as the negative (vehicle) control.

In a laboratory in Poland, the extracts obtained from *P. vulgaris* (PVL—leaves, PVR—roots) were dissolved in DMSO (50 mg/mL) and filtered through a syringe filter (pore diameter 0.2 µm) to obtain stock solutions used in further in vitro studies. Stock solutions were stored frozen until used in experiments.

The evaluation of cytotoxicity was carried out using an MTT microculture tetrazolium assay. Briefly, the VERO and HeLa cells were passaged into 96-well microculture plates in the concentration of 1.5 × 10^5^ cells/mL or 3 × 10^5^ cells/mL, respectively, in growth media. After overnight incubation, the growth media was removed, and serial dilutions of extract stock solution, ranging from 1000 to 0.98 µg/mL, were added, and plates were further incubated for 72 h. The cytotoxicity of DMSO used as a solvent for extract stock solutions was also evaluated. Afterward, all media was removed, the cells were washed with PBS, and 100 µL of cell media supplemented with 10% of MTT solution (5 mg/mL) was added to each well, and incubation continued for the next 4 h. Finally, 100 µL of SDS/DMF/PBS (14% SDS, 36% DMF, 50% PBS) solution per well was added, and after overnight incubation, the absorbance at 540 and 620 nm was measured using Synergy H1 Multi-Mode Microplate Reader (BioTek Instruments, Inc., Winooski, VT, USA) equipped with Gen5 software (ver. 3.09.07, BioTek Instruments, Inc., Winooski, VT, USA) and data were exported to the GraphPad Prism (ver. 8.0.1, GraphPad Software, San Diego, CA, USA) for further analysis. Based on the comparison of absorbance recorded for control and extract-treated cells, values of CC_50_ (concentrations of tested extract decreasing the viability of appropriate cell line by 50%) were calculated [132].

### 3.5. Transfection and Luciferase Assays

HeLa cell cultures bought in ATCC (Bethesda, MD, USA) were maintained in Dulbecco’s modified Eagle’s medium (DMEM) (Gibco Life Technologies, Grand Island, NY, USA) containing 10% fetal bovine serum (FBS, Atlanta Biologicals Inc., Atlanta, GA, USA). HeLa cells were plated in white opaque 384-well plates at a density of 4300 cells/well in 30 µL of growth medium supplemented with 10% FBS and 1% Pen/step. In the next step (the next day), the medium was aspirated and replaced with DMEM containing 10% FBS only. The cells were transfected with respective plasmids using an X-tremeGENE HP transfection reagent (Roche Applied Science, Indianapolis, IN, USA). The following panel of 13 inducible luciferase reporter gene vectors in this assay were used—STAT3, SMAD3/4, NF-κB, AP-1, Ets, E2F, MYC, Notch, FoxO, Wnt, Hedgehog, miR-21, k-Ras. Their expression is driven by enhancer elements (inducer, promoter). Luciferase reporter constructs were transfected into Hela cells [44]. Luciferases are enzymes encoded by genes, which catalyze the bioluminescent reaction of the transformation of the substrate—luciferin, which in the presence of ATP, Mg^2+^ ions, and molecular oxygen is oxidized and becomes excited, and then—returning to the initial state—causes light emission (Figure 4b). The emitted photons are counted with a luminometer. The total light emission is directly proportional to the luciferase activity in the test sample and allows the evaluation of the transcriptional activity of the reporter gene.

After 24 h of transfection, the test agents were added to the transfected cells, followed 30 min later by an inducing agent: IL-6 (50 ng/mL, R&D Systems, Inc., Minneapolis, MN, USA) for Stat3 [44], TGF-beta (5 ng/mL, R&D Systems, Inc., Minneapolis, MN, USA) for Smad [44], Wnt-3a (500 ng/mL, Peprotech Corporation, Rocky Hill, NJ, USA) for Wnt [44] and PMA (phorbol 12-myristate 13-acetate, 77 ng/mL, Sigma Chemical Company, St. Louis, MO, USA) for AP-1, NF-κB, E2F, MYC, Ets, Notch and Hdghog. No inducer was added for FoxO, miR-21, k-Ras, and pTK-control (thymidine kinase promoter). After 4 h (STAT3, SMAD3/4, NF-κB, AP-1, Ets, E2F, MYC) or 6 h (Notch, FoxO, Wnt, Hedgehog, miR-21, k-Ras) of induction, the cells were lysed by the addition of the One-Glo luciferase assay system (Promega Corporation, Madison, WI, USA). A Glomax Multi+ detection system with Instinct Software (Promega Corporation, Madison, WI, USA) was used for the light output detection. This luciferase assay determines if the test agent was able to inhibit the activation of cancer-related signaling pathways [44,88].

## 4. Conclusions

Our research confirmed the strong inhibition of 12 signaling pathways in HeLa cells exerted by methanolic extracts of *P. vulgaris.* In this study, it was found that the root extract of *P. vulgaris* is the most potent in inhibiting the activation of MYC, Notch, Wnt, E2F, Ets, Stat3, Smad, Hdghog, AP-1, NF-κB, k-Ras, miR-21, and pTK-control. This is the first study to report the influence of *Pulsatilla vulgaris* species on cancer signaling pathways. The methanolic extracts of *P. vulgaris* enhanced the apoptotic death of HeLa cells and deregulated cellular proliferation, differentiation, and progression toward the neoplastic phenotype by altering key signaling molecules required for cell cycle progression.

The results showed that *P. vulgaris* is a rich source of triterpenoid saponins and phenolic acids, wherein their triterpenoid saponins showed a different profile in comparison to other species from the Ranunculaceae family. Our research will be helpful to determine the relevance of each cancer-related signaling pathway that may be used to development of novel therapies that combine extracts of *P. vulgaris* with other agents including MYC, Notch, Wnt, E2F, Ets, STAT, SMAD, Hdghog, AP-1, and NF-κB blockers, to effectively treat cervical cancer, and other cancers that activate these pathways.

The study confirms the chemotherapeutic potency of *P. vulgaris* secondary metabolites in different types of cancer line cells and their activity at variable levels of cell signaling. Current trends in oncological pharmacology aim to use multidirectional therapies, which agrees with the pleiotropic character of extracts of *P. vulgaris* inhibiting cancer proliferation in signaling pathways of 12 reporter genes. However, there are still no preclinical and clinical studies that would clearly confirm the benefits of secondary metabolites of *P. vulgaris* in oncological therapy. The problem is the limitation of natural sites of *P. vulgaris* in the EU and the limited bioavailability of substances of natural origin. We need more toxicological studies of extracts of *P. vulgaris* and their secondary metabolites at the cellular, tissue, organ, and organism levels.

## Figures and Tables

**Table 1:** Cytotoxicity of root (PVR) and leaf (PVL) extracts of *Pulsatilla vulgaris* in a panel of normal and cancerous mammalian cell lines tested after 48h incubation using the neutral red assay (normal cell lines: VERO—kidney fibroblast, LLC-PK1—kidney epithelial; cancer cell lines: HeLa—cervical carcinoma, SK-OV-3—ovarian carcinoma, KB—epidermal carcinoma, SK-MEL—skin melanoma, BT-549—breast cancer).

Cytotoxic Activity (CC_50_ μg/mL) ^1^							
Mammalian Cell Lines	Normal Cell Lines		Cancer Cell Lines				
Sample Name	VERO	LLC-PK1	HeLa	SK-OV-3	KB	SK-MEL	BT-549
PVR	39	42	31	35	42	44	57
PVL	52	70	50	60	73	60	68
Doxorubicin ^2^	>5	1.6	3.9	2.3	1.7	1.7	2.2

^1^ CC_50_ μg/mL is the concentration that affords 50% inhibition of cell growth; ^2^—positive control drug; PVR—*P. vulgaris* root extract; PVL—*P. vulgaris* leaves extract.

**Table 2:** The CC_50_ (µg/mL) values obtained for *P. vulgaris* root (PVR) and leaf extracts (PVL) after 72 h incubation using the MTT-based assay.

Extracts	CC_50_^1^ µg/mL (±SD)		Statistical Significance	SI
	VERO	HeLa		
PVR	188.3 ± 7.11	71.87 ± 0.79	**	2.62
PVL	17.57 ± 1.77	21.88 ± 1.73	ns	0.8

^1^ CC_50_—50% cytotoxic concentration; selectivity index (SI)—(CC_50_VERO/CC_50_HeLa); **—statistically highly significant (*p* < 0.001); ns—not significant; PVR—*P. vulgaris* root extract; PVL—*P. vulgaris* leaves extract.

## Data Availability

Not applicable.

## Supplementary Materials

The following supporting information can be downloaded at: https://www.mdpi.com/article/10.3390/ijms24021139/s1.

## Abbreviations

Transcription factors—Stat3, Smad, AP-1, NF-κB, E2F, MYC, Notch, Wnt, Hdghog, Ras; inducers—cytokines IL-6, ligand TGF-beta, PMA—protein kinase activator, phorbol 12-myristate-13-acetate, Wnt-3a protein; Ser/Thr—serine/threonine kinases, EGFR—epidermal growth factor receptor, STAT—signal transducer and activator of the transcription protein family, MEKK—mitogen-activated protein (MAP) kinase, NOTCH—neurogenic locus notch homolog protein, IkB—inhibitor of kB, JAK—Janus kinase, Src—protooncogene tyrosine-protein kinase, Rac—subfamily of the Rho family of GTPases, Ras—Ras family kinase, p38—mitogen-activated protein kinases, p15, p16—the cyclin-dependent kinase inhibitors, CDKs—cyclins, cyclin-dependent kinases, Patched—protein patched homolog.
