# Supplementary material for: Pulsatilla vulgaris Inhibits Cancer Proliferation in Signaling Pathways of 12 Reporter Genes
Source: Int J Mol Sci. 2023 Jan 6;24(2):1139. doi: 10.3390/ijms24021139 (PMC9860614; doi:10.3390/ijms24021139)
Supplement: Supplementary file 1 [file ijms-24-01139-s001.zip › ijms-2091305-supplementary.pdf]

Supplementary material

Pulsatilla vulgaris Inhibits Cancer Proliferation in Signaling Pathways of 12 Reporter Genes

Grażyna Łaska [1], Elwira Sieniawska [2,*], Magdalena Maciejewska-Turska [3], Łukasz Świątek [4], David S. Pasco [5] and Premalatha Balachandran [5]

[1] Department of Agri-Food Engineering and Environmental Management, Bialystok University of Technology, 15-351 Bialystok, Poland
[2] Department of Natural Products Chemistry, Medical University of Lublin, 20-093 Lublin, Poland
[3] Department of Pharmacognosy with the Medicinal Plant Garden, Medical University of Lublin, 20-093 Lublin, Poland
[4] Department of Virology with SARS Laboratory, Medical University of Lublin, 20-093 Lublin, Poland
[5] National Center for Natural Products Research, Research Institute of Pharmaceutical Sciences, School of Pharmacy, University of Mississippi, University, MS 38677, USA
* Correspondence: esieniawska@pharmacognosy.org

Supplementary Table S1. Results of tests of biological activity of secondary metabolites isolated from methanolic extract of roots (mR) and leaves (mL) of *Pulsatilla vulgaris* Mill. and reference/control compound - resveratrol analogue, in the presence of various inducers on human cervical carcinoma HeLa cell lines with 13 different vectors (transcription factors) in volume 40 μg/mL (I assay)

	**Transcription factors (vectors)[1] in the presence of various inducers [2] in the luciferase reporter gene assay**													
**Sample data**	**Stat3/ IL-6**	**Smad/ TGF-β**	**AP-1/ PMA**	**NF-κB/ PMA**	**E2F/ PMA**	**MYC/ PMA**	**Ets/ PMA**	**Notch (CSL-Luc)**	**FoxO in 10% FBS**	**Wnt/ wnt-3a**	**Hdghog/ PMA**	**pTK (4h)**	**miR-21**	**k-Ras**
**Resveratrol analog (6 μM)**	92[3]	68	78	28	64	47	62	65	66	44	57	95	78	86
***P. vulgaris* (mL 40 μg/mL) - I assay**	98	154	96	57	115	96	106	109	134	179	120	141	156	126
***P. vulgaris* (mR 40 μg/mL) - I assay**	-6	-4	-1	0	-11	-37	-7	-31	132	-13	-4	2	2	1

Explanations to the table:

[1] **Transcription factors, vectors in the signal transduction pathways of cancer processes** - Stat3, Smad, AP-1, NF-κB, E2F, MYC, Ets, Notch, FoxO, Wnt, Hdghog, miR-21, k-Ras and pTK - control

[2] **Inducer, promotor** of cancer processes - IL-6, TGF-β, PMA, wnt-3a

[3] Numerical value expressing the percentage ratio of the activity of the tested samples (metabolites) to the activity of the promoter based on the determination of the activity of the protein encoded by the luciferase reporter gene.
The lower the numerical value in the table, the greater the activity of the test sample is in inhibition than that of the tumor promoter.

Resveratrol analog – compounds with antitumor activity used in the research (explanations in the text)
P. vulgaris (mL 40 μg/mL) – is the volume of methanolic extract from the leaves of the *P. vulgaris* used for the -I assay - not biologically active
P. vulgaris (mR 40 μg/mL) – is the volume of methanolic extract from the root of the *P. vulgaris* used for the – I assay - biologically active antitumor

Supplementary Table S2. Results of tests of biological activity of secondary metabolites isolated from methanolic extract of roots (mR) of *Pulsatilla vulgaris* Mill. using their different volumes: 40 µg/mL (I assay) and 15 µg/mL and 10 µg/mL (II assay), in the presence of various inducers on human cervical carcinoma HeLa cell lines with 13 different vectors (transcription factors)

	**Transcription factors (vectors)[1] in the presence of various inducers [2] in the luciferase reporter gene assay**													
**Sample data**	**Stat3/ IL-6**	**Smad/ TGF-β**	**AP-1/ PMA**	**NF-κB/ PMA**	**E2F/ PMA**	**MYC/ PMA**	**Ets/ PMA**	**Notch (CSL-Luc)**	**FoxO in 10% FBS**	**Wnt/ wnt-3a**	**Hdghog/ PMA**	**pTK (4h)**	**miR-21**	**k-Ras**
**Resveratrol analog (6 µM)**	92[3]	68	78	28	64	47	62	65	66	44	57	95	78	86
***P. vulgaris* (mR 40 µg/mL) - I assay**	-6	-4	-1	0	-11	-37	-7	-31	132	-13	-4	2	2	1
***P. vulgaris* (mR 15 µg/mL) - II assay**	-5	-4	-1	-9	-6	-33	-14	-31	129	-20	-4	4	8	2
***P. vulgaris* (mR 10 µg/mL) - II assay**	36	9	95	11	99	17	23	120	126	34	88	105	168	113

Explanations to the table:

[1] **Transcription factors, vectors in the signal transduction pathways of cancer processes** - Stat3, Smad, AP-1, NF-κB, E2F, MYC, Ets, Notch, FoxO, Wnt, Hdghog, miR-21, k-Ras and pTK - control

[2] **Inducer, promotor** of cancer processes - IL-6, TGF-β, PMA, wnt-3a

[3] Numerical value expressing the percentage ratio of the activity of the tested samples (metabolites) to the activity of the promoter based on the determination of the activity of the protein encoded by the luciferase reporter gene.
The lower the numerical value in the table, the greater the activity of the test sample is in inhibition than that of the tumor promoter.

Resveratrol analog – compounds with antitumor activity used in the research (explanations in the text)
P. vulgaris (mR 40 µg/mL) – is the volume of methanolic extract from the root of the *P. vulgaris* used for the tests - test I - biologically active antitumor
P. vulgaris (mR 15 µg/mL) – is the volume of methanolic extract from the root of the *P. vulgaris* used for the tests - test II - biologically active antitumor
P. vulgaris (mR 10 µg/mL) – is the volume of methanolic extract from the root of the *P. vulgaris* used for the tests - test II - biologically active antitumor
